# The m6A Methyltransferase METTL3-Mediated N6-Methyladenosine Modification of DEK mRNA to Promote Gastric Cancer Cell Growth and Metastasis

**DOI:** 10.3390/ijms23126451

**Published:** 2022-06-09

**Authors:** Hui-Min Zhang, Fei-Fei Qi, Jun Wang, Yuan-Yuan Duan, Li-Li Zhao, Yun-Dan Wang, Tong-Cun Zhang, Xing-Hua Liao

**Affiliations:** Institute of Biology and Medicine, College of Life and Health Sciences, Wuhan University of Science and Technology, Wuhan 430000, China; huiminzhang@wust.edu.cn (H.-M.Z.); qifeifei@wust.edu.cn (F.-F.Q.); 201818703005@wust.edu.cn (J.W.); a1662687624@126.com (Y.-Y.D.); lilizhao2022@126.com (L.-L.Z.); yundanwang@wust.edu.cn (Y.-D.W.)

**Keywords:** m6A methylation modification, METTL3, DEK, gastric cancer (GC), cell proliferation, cell migration

## Abstract

Gastric cancer (GC) is the fifth most common cancer and the third deadliest cancer in the world, and the occurrence and development of GC are influenced by epigenetics. Methyltransferase-like 3 (METTL3) is a prominent RNA n6-adenosine methyltransferase (m6A) that plays an important role in tumor growth by controlling the work of RNA. This study aimed to reveal the biological function and molecular mechanism of METTL3 in GC. The expression level of METTL3 in GC tissues and cells was detected by qPCR, Western blot and immunohistochemistry, and the expression level and prognosis of METTL3 were predicted in public databases. CCK-8, colony formation, transwell and wound healing assays were used to study the effect of METTL3 on GC cell proliferation and migration. In addition, the enrichment effect of METTL3 on DEK mRNA was detected by the RIP experiment, the m6A modification effect of METTL3 on DEK was verified by the MeRIP experiment and the mRNA half-life of DEK when METTL3 was overexpressed was detected. The dot blot assay detects m6A modification at the mRNA level. The effect of METTL3 on cell migration ability in vivo was examined by tail vein injection of luciferase-labeled cells. The experimental results showed that METTL3 was highly expressed in GC tissues and cells, and the high expression of METTL3 was associated with a poor prognosis. In addition, the m6A modification level of mRNA was higher in GC tissues and GC cell lines. Overexpression of METTL3 in MGC80-3 cells and AGS promoted cell proliferation and migration, while the knockdown of METTL3 inhibited cell proliferation and migration. The results of in vitro rescue experiments showed that the knockdown of DEK reversed the promoting effects of METTL3 on cell proliferation and migration. In vivo experiments showed that the knockdown of DEK reversed the increase in lung metastases caused by the overexpression of METTL3 in mice. Mechanistically, the results of the RIP experiment showed that METTL3 could enrich DEK mRNA, and the results of the MePIP and RNA half-life experiments indicated that METTL3 binds to the 3’UTR of DEK, participates in the m6A modification of DEK and promotes the stability of DEK mRNA. Ultimately, we concluded that METTL3 promotes GC cell proliferation and migration by stabilizing DEK mRNA expression. Therefore, METTL3 is a potential biomarker for GC prognosis and a therapeutic target.

## 1. Introduction

According to the latest report of the International Agency for Research on Cancer, GC (GC) is the fifth most common cancer and the third deadliest cancer worldwide [1]. The bad incidence, prognosis and cellular and molecular heterogeneity of GC make this disease a major health problem worldwide [2]. At present, the application of gene-targeted therapy is gradually increasing, and oncogenes and tumor suppressor genes play an important role in the targeted therapy of GC. In addition, the occurrence and development of GC are affected by epigenetics [3]. Therefore, it is of great significance to study the molecular mechanism of the occurrence and development of GC.

N6-methyladenosine (m6A) is the most abundant internal mRNA modification [4], and at present, m6A has become a widespread regulatory mechanism for regulating gene expression [5]. Cancer initiation and progression often result in the derangement of genomic and epigenetic regulation, which frequently results in the aberrant expression of a core set of genes associated with persistent proliferation, disruption of apoptosis, abnormal stemness and treatment resistance [6]. There is increasing evidence that m6A methyltransferases cause many cancers due to their ability to determine the fate of RNA [5]. Furthermore, the ablation of m6A methyltransferase has been shown to affect tumor progression [7]. These observations suggest that catalyzing m6A modifications of key genes involved in cancer biology may be critical for their function. There are three methylases in m6A methylation: methyltransferase-like 3 (METTL3), methyltransferase-like 14 (METTL14) and Wilms tumor 1-associated protein (WTAP) [8]. METTL3 has been shown to be significantly overexpressed in lung cancer, liver cancer and GC, and involved in regulating tumor progression [9,10,11]. However, the mechanism of METTL3 in the development of gastric cancer is still inconclusive.

As a nuclear protein, DEK is involved in various nuclear processes such as DNA replication, RNA splicing, transcriptional regulation and DNA repair through chromatin organization and nucleic acid binding [12]. Recently, overexpression of DEK was found to be positively correlated with tumorigenesis and metastasis in various cancers [13,14]. Although DEK has been shown to be overexpressed in gastric adenocarcinoma [15], the detailed mechanisms of how DEK is regulated in GC and how DEK affects the disease remain largely unclear. This study showed that METTL3 promoted the proliferation and metastasis of GC by modifying DEK mRNA m6A.

## 2. Results

### 2.1. METTL3 Is Highly Expressed in GC

**Figure 1:**
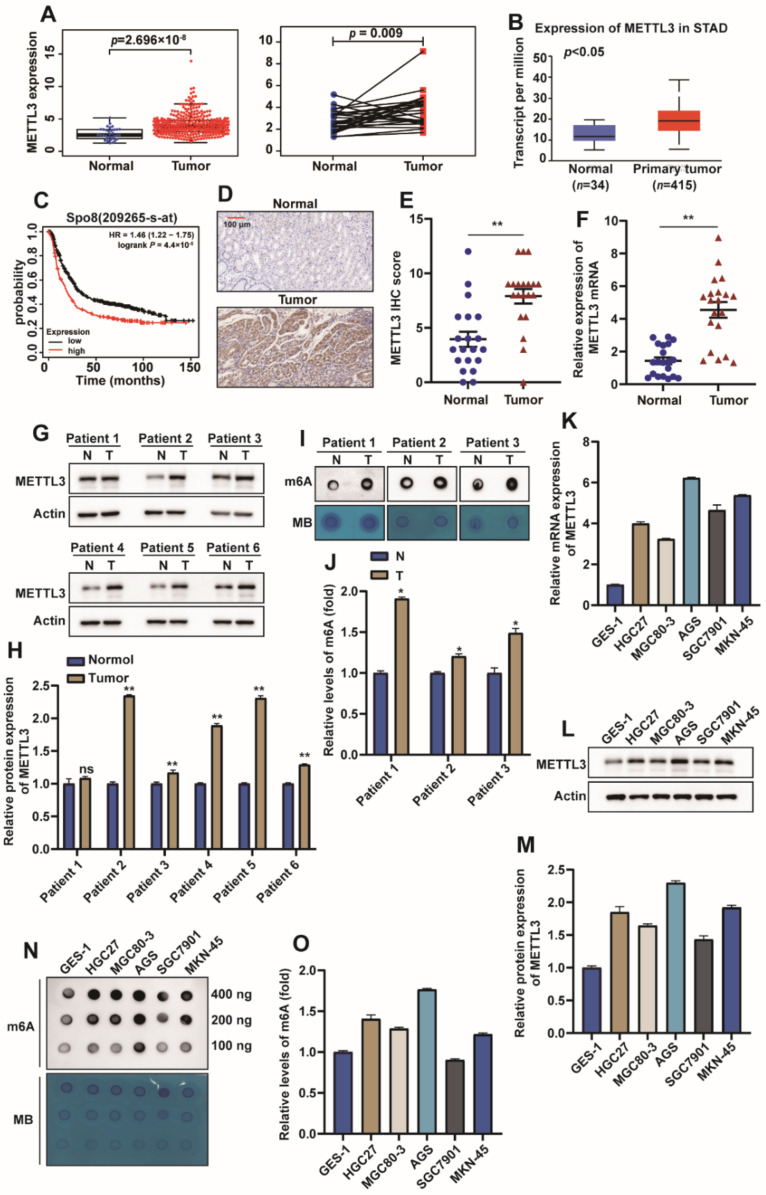
METTL3 is highly expressed in GC. (**A**) The expression levels of METTL3 in gastric cancer tissues and normal tissues were analyzed in the TCGA database. (**B**) The expression level of METTL3 in gastric cancer was analyzed by UALCAN. (**C**) The relationship between the expression level of METTL3 and prognosis was analyzed in the TCGA database. (**D**) Representative images of METTL3 immunohistochemistry (IHC) of normal GC tissue (left) and GC tissue (right) (scar bar = 100 µm). (**E**) Calculated GGCT immunohistochemical scores in GC group (*n* = 20) and normal group (*n* = 20). (**F**) The mRNA level of METTL3 was detected by qPCR in GC normal tissues and cancer tissues, and Actin was used as an internal control. (**G**,**H**) The protein expression level of METTL3 in GC tissues and normal tissues was detected by Western blot, and the gray value of the experimental results was analyzed and normalized by Actin (*n* = 3). (**I**,**J**) The m6A modification level of mRNA in GC tissue and normal tissue was detected by dot blot experiment, in which the total amount of RNA used in each spot was 400 ng, and the experimental results were quantitatively analyzed. (**K**) qPCR detection of METTL3 mRNA levels in human gastric mucosa normal epithelial cells (GES1) and cancer cells (MGC80-3, HGC27, AGS, SGC7901, MKN-45); Actin was used as an internal control (*n* = 3). (**L**,**M**) The protein levels of METTL3 in human gastric mucosa normal epithelial cells (GES1) and cancer cells (MGC80-3, HGC27, AGS, SGC7901, MKN-45) were detected by Western blot, and were normalized with Actin (*n* = 3). (**N**,**O**) Dot blot assay to detect the m6A modification level of mRNA in GES1, MGC80-3, HGC27, AGS, SGC7901, MKN-45 cells, and the dot blot results for 100 ng, 200 ng and 400 ng RNA amounts were quantified separately and compared to GES-1 cells, then averaged and counted (*n* = 3). (*: *p* < 0.05, **: *p* < 0.01).

In order to explore the potential significance of m6A modification in GC, the expression level of METTL3 in GC was investigated. TCGA database analysis showed that METTL3 in tumor tissue was significantly higher than that in normal tissue in both unpaired (Figure 1A left) and paired (Figure 1A right) sample comparisons and consistent with UALCAN (http://ualcan.path.uab.edu/cgi-bin/ualcan-res.pl accessed on 20 September 2021) website analysis results (Figure 1B). Meanwhile, TCGA database data also indicated that the high expression of METTL3 was associated with poor prognosis in GC patients (Figure 1C). Next, immunohistochemical analysis was performed on cancer tissues and normal tissues of 20 patients, and the immunohistochemical results were scored. The results showed that METTL3 was mainly expressed in the nucleus and was highly expressed in GC tissues (Figure 1D,E). Then, the mRNA and protein expression levels of METTL3 in GC tissues were analyzed by qPCR and Western blot, respectively, and the results of Western blot were quantified. The experimental results showed that the mRNA and protein levels of METTL3 in GC tissues were higher than those in normal tissues (Figure 1F–H). We further divided patients into low expression groups (*n* = 10) and high expression groups (*n* = 10) according to the median expression level of METTL3. The correlation between METTL3 expression level and clinicopathological characteristics such as age, gender, TNM stage, distant metastasis and lymph node metastasis of gastric cancer patients is shown in Supplementary Table S1. The METTL3 high expression group was associated with advanced TNM and higher degrees of distant metastasis and lymph node metastasis (*p* < 0.05). However, there was no significant correlation between METTL3 level and patient gender and age (*p* > 0.05). Evidence accumulated in recent years suggests that METTL3 as an m6A methyltransferase plays a key role in cancer, both as an oncogene and as a tumor suppressor [16,17]. The m6A mRNA modification level in the patient tissues was detected by m6A RNA dot blot assay, and the results were quantified. The results showed that the m6A mRNA modification level in GC tissues was high (Figure 1I,J). We further verified the high expression of METTL3 at the cellular level. RNA and protein were extracted from normal gastric epithelial cells (GES1) and GC cells (HGC27, MGC80-3, AGS, SGC7901, MKN-45). qPCR and Western blot results showed that METTL3 was highly expressed in GC cell lines (Figure 1K,L). Quantification of Western blot results also confirmed this conclusion (Figure 1M). The m6A RNA dot blot assay also showed that the m6A mRNA modification level in GC cells was increased (Figure 1N,O). The above results indicate that METTL3 is highly expressed in GC tissues and cells, and the high expression of METTL3 is associated with a poor prognosis of GC. Meanwhile, m6A is highly modified in GC tissues and cells.

### 2.2. METTL3 Promotes the Proliferation and Migration of GC Cells

**Figure 2:**
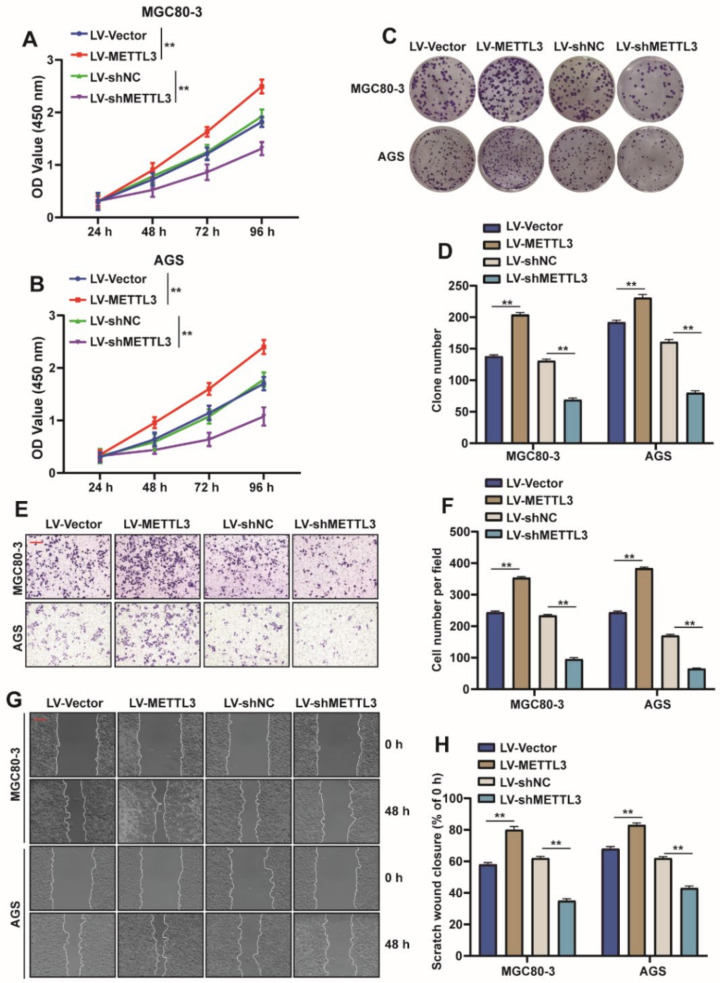
METTL3 promotes the proliferation and migration of GC cells. (**A**,**B**) Lentivirus-introduced CDS or shMETTL3, CCK-8 of METTL3 was used in MGC80-3 and AGS cells to detect cell viability (*n* = 3, **: *p* < 0.01). (**C**,**D**) Cell cloning assays to assess the proliferation capacity of MGC80-3 and AGS cells when METTL3 was overexpressed or knocked down, and the number of cell clones was statistically analyzed (*n* = 3, **: *p* < 0.01). (**E**,**F**) Transwell assays assessed the migratory ability of MGC803 and AGS cells when METTL3 was overexpressed or knocked down, and the number of migrated cells was statistically analyzed (*n* = 3, **: *p* < 0.01). (**G**,**H**) Wound healing assays to evaluate the migratory ability of MGC803 and AGS cells when METTL3 was overexpressed or knocked down, and statistical analysis of wound healing ability was performed (*n* = 3, **: *p* < 0.01).

Since METTL3 is highly expressed in GC and is associated with poor prognosis of patients with GC, an assumption was made that METTL3 could perform the function of tumor promoter in GC. In order to explore the function of METTL3, METTL3 was overexpressed or knocked down in MGC80-3 and AGS cells. As expected, METTL3 overexpression significantly promoted cell viability and the proliferation of GC cells, as indicated by the increased cell growth rate (Figure 2A,B) and increased colony number (Figure 2C,D), whereas the effect was reversed when METTL3 was knocked down. Further, the transwell experiment showed that overexpression of METTL3 promoted the migration of GC cells, while knockdown of METTL3 inhibited the migration ability of GC cells (Figure 2E,F), which was further confirmed by the wound healing experiment (Figure 2G,H). The above experimental data suggest that METTL3 plays a role in promoting cell proliferation and migration in GC cells.

### 2.3. METTL3 Regulated DEK Expression in GC

**Figure 3:**
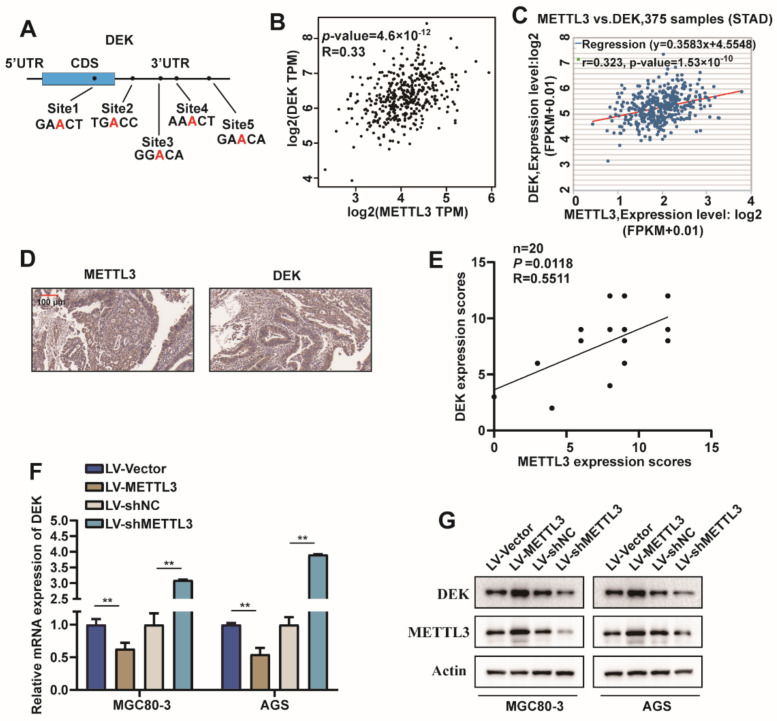
METTL3 regulated DEK expression in GC. (**A**) DEK m6A modification site prediction pattern map predicted by m6A2Target and SRAMP database (Both pre-mRNA genomic sequences (with introns) and mature mRNA sequences (containing 3- and 5-terminal non-coding sequences) of DEK were predicted at the SRAMP site. Site 1 was not predicted from the pre-mRNA genome sequence). (**B**) The GEPIA database predicts the correlation of METTL3 with DEK expression. (**C**) The ENCORI website predicts METTL3 in association with DEK expression. (**D**) Representative images of METTL3 and DEK immunohistochemical staining in tumor sections from the same patient. (**E**) The graph shows the correlation of METTL3 with DEK IHC scores and gives the Pearson correlation coefficient (R), *p*-value, as well as simple numbers (*n*) in the upper corner. (**F**,**G**) METTL3 was overexpressed or knocked down in MGC80-3 and AGS cells, DEK mRNA levels were detected by qPCR, and protein levels were detected by Western blot (*n* = 3, **: *p* < 0.01).

To further investigate the mechanism of action of METTL3 in GC proliferation and migration, possible modified substrates were first predicted in the m6A2Target database. Additionally, the possible modification sites of DEK were predicted on the SRAMP website (Figure 3A). However, the relationship between METTL3 and DEK in GC is not clear. Therefore, we analyzed the correlation between METTL3 and DEK in the GEPIA database, and the results showed that METTL3 was positively correlated with DEK expression (Figure 3B), which was consistent with the results of ENCORI website analysis (Figure 3C). Furthermore, to evaluate the relationship between METTL3 and DEK expression, we carried out IHC analyses of human GC samples and observed that METTL3 was positively correlated with DEK protein levels (Figure 3D,E). It was further found that overexpression of METTL3 in MGC80-3 and AGS cells promoted DEK mRNA and protein expression, whereas the knockdown of METTL3 had the opposite result (Figure 3F,G). Collectively, these results suggested METTL3 is a critical m6A methylase that regulates DEK expression in GC.

### 2.4. METTL3 Stabilize DEK mRNA via m6A Modification in GC

**Figure 4:**
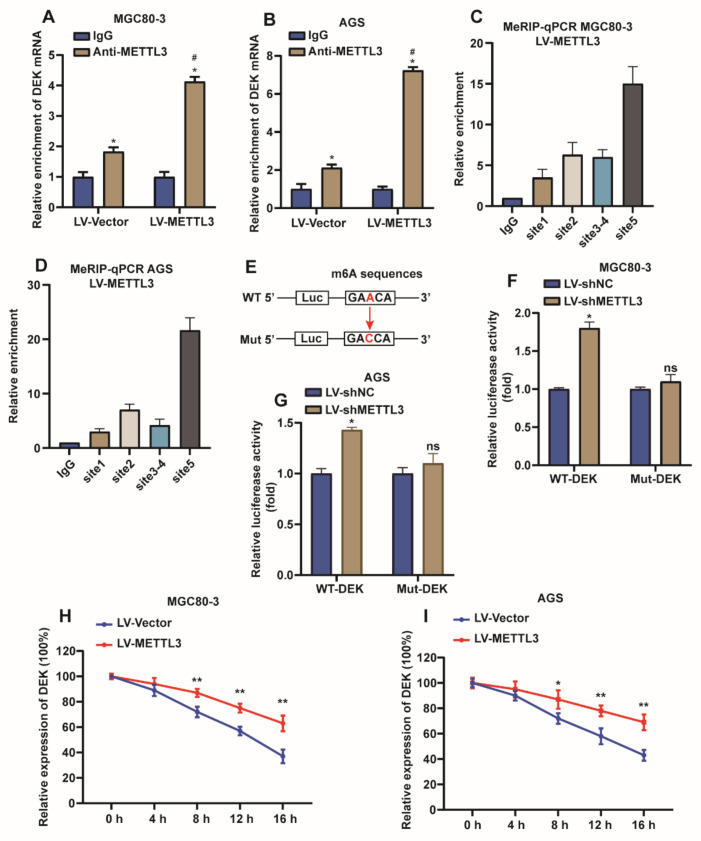
METTL3 stabilize DEK mRNA via m6A modification in GC. (**A**,**B**) RIP-qPCR assays were used to detect the enrichment of METTL3 binding DEK in MGC80-3 and AGS cells (*, vs. IgG, *p* < 0.05; #, vs. anti-METTL3 in NC group, *p* < 0.05). (**C**,**D**) MeRIP-qPCR assay assessing m6A enrichment of DEK mRNA when METTL3 was overexpressed in MGC80-3 and AGS cells (*n* = 3, **: *p* < 0.01). (**E**) Luciferase reporters of wild type and mutation DEK were constructed. (**F**,**G**) Mutation of the m6A recognition sequence or knockdown of METTL3 increased luciferase activity in MGC80-3 and AGS cells (*n* = 3, *: *p* < 0.05). (**H**,**I**) METTL3 was overexpressed in MGC80-3 and AGS cells, and the half-life of DEK mRNA was detected by qPCR (*n* = 3, **: *p* < 0.01).

For METTL3 targets DEK mRNA via m6A modification verification, METTL3 was overexpressed in MGC80-3 and HGC27 cells, and then the enrichment effect of METTL3 on DEK mRNA was detected by RIP assay. We found that METTL3 enriched DEK mRNA, and this enrichment was more pronounced when METTL3 was overexpressed (Figure 4A,B). Further, MeRIP-qPCR experiments were used to examine the modification of DEK mRNA by m6A when METTL3 was overexpressed in MGC80-3 and AGS cells. The results showed that compared with the IgG group, DEK had m6A modification, and the modification sites were mainly concentrated in site 5 (Figure 4C,D) (the same pair of primers were used for site 3 and site 4 because the difference was within 50 bp). To further determine the site of m6A modification of DEK mRNA, we used a luciferase reporter gene containing wild-type or mutant DEK to study the m6A modification of DEK mRNA. In DEK mutants, the adenosine group in the m6A recognition sequence is replaced by cytosine, thereby eliminating the m6A modification (Figure 4E). The results of the luciferase reporter assay showed that the knockdown of METTL3 promoted luciferase activity in the WT-DEK group but had no effect on the Mut-DEK group (Figure 4F,G). In addition, we measured the loss of DEK mRNA in MGC80-3 and AGS cells treated with α-amanitin, an inhibitor of RNA synthesis. For MGC80-3 and AGS cells, the mRNA of DEK in the METTL3-overexpressing group still did not reach the half-life at 16 h, but the half-life was reached between 12–16 h in the control group (MGC80-3:t1/2 = 13.4 h, AGS: t1/2 = 14.1 h). This indicates that overexpression of METTL3 can prolong the half-life of DEK mRNA in MGC80-3 and AGS cells (Figure 4H,I). In conclusion, METTL3 positively regulates DEK expression by recognizing the m6A modification site in the 3’UTR region of DEK mRNA.

### 2.5. METTL3 Promotes Proliferation and Migration of GC Cells by Regulating DEK

**Figure 5:**
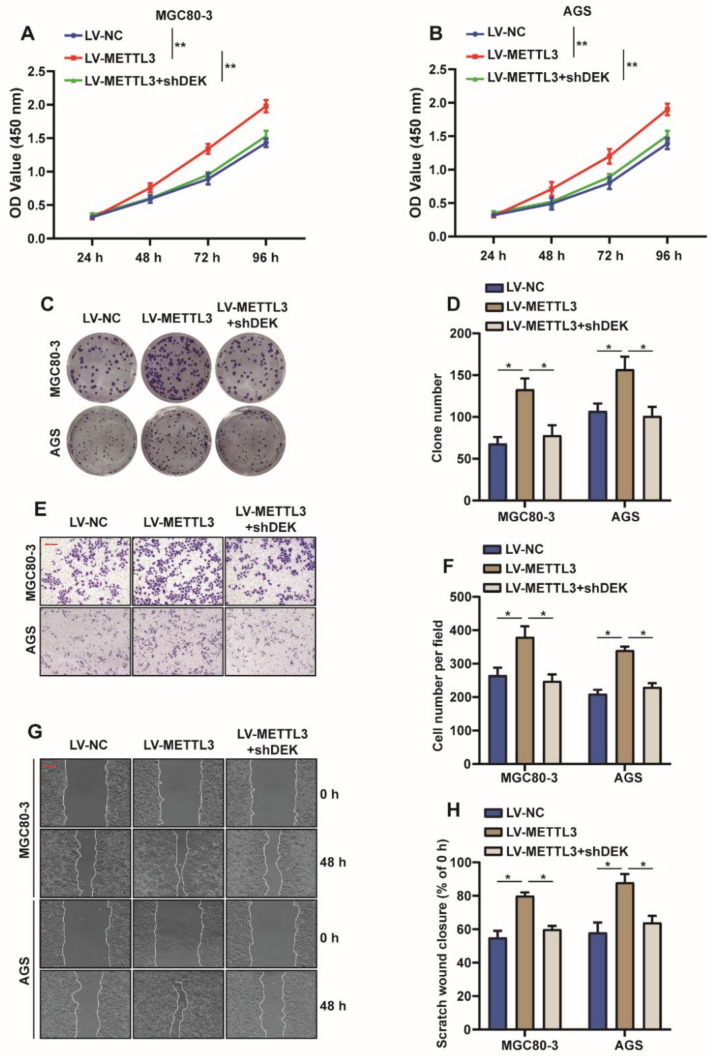
METTL3 promotes proliferation and migration of GC cells by regulating DEK. (**A**,**B**) Overexpression of METTL3 in MGC80-3 and AGS cells increased cell viability, and knockdown of DEK when METTL3 was overexpressed reversed the effect of METTL3 on cell viability (*n* = 3, **: *p* < 0.01). (**C**,**D**) Overexpression of METTL3 in MGC80-3 and AGS cells promotes cell clone formation, and knockdown of DEK when METTL3 is overexpressed reverses the effect of METTL3 on clone formation (*n* = 3, *: *p* < 0.05). (**E**,**F**) Overexpression of METTL3 in MGC80-3 and AGS cells promoted cell migration and knockdown of DEK when METTL3 was overexpressed reversed the promotion of METTL3 on cell migration (*n* = 3, *: *p* < 0.05). (**G**,**H**) Overexpression of METTL3 in MGC80-3 and AGS cells promotes cellular wound healing, and knockdown of DEK when METTL3 is overexpressed reverses the effect of METTL3 on cellular wound healing (*n* = 3, *: *p* < 0.05).

Based on the above studies, we concluded that METTL3 is involved in the m6A modification of DEK and affects the expression of DEK. However, it is unclear whether METTLE regulates the proliferation and migration of GC cells by affecting DEK expression. Therefore, a series of rescue experiments were carried out. We overexpressed METTL3 and knocked down DEK in MGC80-3 and AGS cells and found that the knockdown of DEK reversed the enhanced cell viability and increased cell clones caused by overexpression of METTL3 (Figure 5A–D). At the same time, transwell and wound healing experiments suggested that the knockdown of DEK antagonized the enhanced cell migration ability caused by the overexpression of METTL3 (Figure 5E–H). Taken together, it is further confirmed that METTL3 promotes the proliferation and migration of GC cells by regulating DEK.

### 2.6. METTL3 Promotes GC Metastasis In Vivo

**Figure 6:**
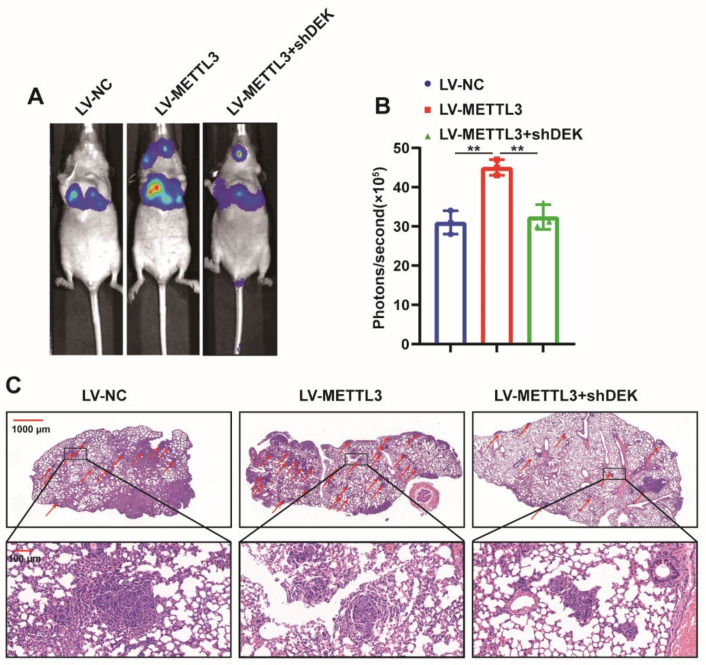
METTL3 promotes GC metastasis in vivo. (**A**) LV-NC, LV-METTL3 and LV-METTL3+shDEK cells were injected into nude mice via tail vein, and the growth of lung metastases was monitored by bioluminescence imaging 28 days later (*n* = 3). (**B**) Statistical analysis of luminescence values of nude mice bioluminescence imaging to monitor lung metastases (**: *p* < 0.01). (**C**) Representative image of nude mouse lung tissue for HE staining to evaluate lung metastases.

In order to further explore the role of METTL3 in GC, luciferase-labeled MGC80-3 cells were constructed (MGC80-3-Luc), then METTL3 or shDEK was introduced into MGC80-3-Luc cells with lentivirus and finally, the cells were injected into nude mice via the tail vein, and the growth of lung metastases was monitored by bioluminescence imaging (BLI). The results indicated that METTL3 promoted lung metastasis of GC cells, while the knockdown of DEK antagonized the promotion of METTL3 on the growth of GC cell metastases (Figure 6A,B). HE staining of mouse lung tissue showed that the number of lung metastases in the METTL3 overexpression group was significantly increased compared with that in the control group. In contrast, in the overexpression of METTL3 and introduction of shDEK, the lung metastases were less than in the overexpression of METTL3 alone (Figure 6C). The above results further confirmed the role of METTL3 as a tumor suppressor gene in GC growth and metastasis in vivo.

## 3. Discussion

In this study, we found that METTL3 is highly expressed in gastric cancer, and the high expression of METTL3 is associated with the poor prognosis of patients. Overexpression of METTL3 promoted the proliferation and migration of gastric cancer cells, while the knockdown of METTL3 had the opposite effect. In addition, the knockdown of DEK was found to reverse the effects of METTL3 on the proliferation and migration ability of gastric cancer cells, which was further verified by a lung metastasis model at the animal level. Mechanistically, METTL3 enriches DEK mRNA, modifies DEK by m6A by binding to DEK 3’UTR and promotes DEK expression by stabilizing DEK mRNA.

GC is the fifth most common malignant tumor in the world. It is characterized by high mortality, a high degree of malignancy and strong heterogeneity [18]. Invasion and metastasis are common features of advanced GC, leading to poor prognosis in GC patients [19]. Metastasis is associated with poor prognosis and high mortality in cancer patients. Therefore, understanding the pathological mechanisms, especially proliferation and metastasis, is critical to direct GC therapy. Although non-coding RNAs have important contributions to cell–cell communication, revealing the complex interactions between tumor cells, tumor microenvironment cells and immune cells, and analyzing their content in body fluids have become mainstream in biomarker identification [20], increasing evidence suggests that alterations in epigenetic mechanisms can guide cancer onset and progression, such as DNA methylation, histone methylation modifications and histone acetylation [21]. In recent years, researchers identified m6A RNA modifications as epigenetic regulators involved in the dynamic and reversible control of RNA structure and function in tumors, including colorectal, liver, breast, nasopharyngeal and gastric cancers [22,23,24]. Prominent evidence in the study further suggests that m6A methylation is a central regulator of human cancer pathogenesis [25].

METTL3 is a key component of the large m6A methyltransferase complex in mammals, responsible for the modification of m6A in various RNAs [26]. Elevated METTL3 is associated with poor prognosis in GC patients [27,28]. It was reported that METTL3 modifies ZMYM1 mRNA with m6A and enhances its stability and expression in GC. ZMYM1 then recruits the CTBP/LSD1/COREST complex to bind to the E-cadherin promoter and repress its expression, thereby promoting EMT and GC transfer [11]. In addition, METTL3 promotes the resistance of CD133+ GC stem cells to oxaliplatin by promoting the stability of PARP1 mRNA and increasing the activity of the basal excision repair pathway [29]. Huo et al. found that METTL3 promotes the translation of SPHK2 mRNA in an m6A-YTHF1-dependent manner. SPHK2 functionally promotes the proliferation, migration and invasion of GC cells by inhibiting the expression of KLF2 [30]. The finding that METTL3 promotes the proliferation and metastasis of GC through m6A modification of the YAP1 pathway was also reported [31]. METTL3 is upregulated in most tumors, including nasopharyngeal [32], gastric [11], liver cancer [33] and bladder cancer [34], but it is downregulated in colorectal cancer [35]. The differential expression of METTL3 across tumors has a dual role, which may reflect differences in targeting pathways and tumor heterogeneity. Our study found that METTL3 was highly expressed in GC tissues and cells, and METTL3 enhanced the mRNA stability of DEK through the m6A modification of DEK, which further promoted the expression of DEK. In conclusion, METTL3 promotes cell proliferation and migration through DEK.

DEK has been identified as an oncogene that regulates cellular processes in many types of cancer. DEK is highly expressed in breast cancer and is associated with malignant phenotype and progression [36]. DEK-induced cytokine dysregulation is associated with M2 macrophage (TAM) polarization, creating a potentially pro-tumor microenvironment through M2 polarization of tumor-associated macrophages [37]. Additionally, DEK is overexpressed in high-grade serous ovarian cancers (HGSOCs), and reducing DEK levels significantly reduces cell viability and tumor growth, resulting in apoptotic cell death [38]. Cai et al. showed that Gigantol inhibits cell proliferation and induces apoptosis by regulating DEK in non-small cell lung cancer [39]. In the report on GC, Lee et al. reported that DEK is a potential biomarker associated with malignant phenotype in GC Tissues and Plasma [40], and Fan et al. demonstrated that circ_0000039 upregulates DEK expression by adsorbing miR-1292-5p, promoting the proliferation and progression of GC cells [41]. Zhang et al. found that overexpression of miR-138-5p inhibited the proliferation and migration of GC cells, increased apoptosis and inhibited tumor growth. However, overexpression of DEK abrogated miR-138-5p overexpression-mediated inhibition of GC cell proliferation and cell cycle arrest [42]. In addition, Hui et al. discovered that MicroRNA-1292-5p inhibits cell growth, migration and invasion of gastric carcinoma by targeting DEK [43]. Our previous study showed that DEK inhibits apoptosis and promotes autophagy in gastric cancer [44], so we aimed at the molecular mechanism by which DEK exerts a tumor-promoting effect in gastric cancer. In this study, we found that METTL3 promoted the proliferation and migration of gastric cancer cells, and the knockdown of DEK reversed the effect of METTL3 on the proliferation and migration of gastric cancer cells. Further studies showed that METTL3 was involved in the m6A modification of DEK mRNA, improving the stability of DEK mRNA and promoting the expression of DEK. In conclusion, our study found that METTL3 promoted cell proliferation and migration via DEK.

## 4. Materials and Methods

### 4.1. Tissue Samples

Tongji Hospital of Huazhong University of Science and Technology provided human GC and adjacent benign tissues (*n* = 20). All patients did not receive radiotherapy or chemotherapy before surgery. All procedures performed in studies involving human participants were in accordance with the 1964 Declaration of Helsinki and its subsequent amendments or similar ethical standards, all participants gave informed consent and the study protocol was approved by the Ethics Committee of Tongji Hospital, Huazhong University of Science and Technology (the approval number IRB ID: TJ-C20210310. Date: 21 March 2021).

### 4.2. Cell Lines

Human gastric mucosa epithelial cells GES1, human gastric cancer cells MGC-803, AGS, MKN-45 and HGC27 cells were purchased from Beina Bio (BNCC, Beijing, China). MGC-803, MKN-45 and HGC27 cells were reared in RPMI-1640 complete medium (Meilunbio, Dalian, China). AGS cells were fed in F-12 complete medium (Meilunbio, Dalian, China) and GES1 in DMEM-H complete medium (Meilunbio, Dalian, China). All media contained 10% fetal bovine serum (FBS) (Gibco, New York, NY, USA) and 1% penicillin-streptomycin (Beyotime, Shanghai, China), and all cells were grown in an incubator at 37 °C and 5% CO_2_.

### 4.3. Quantitative Real-Time PCR (qRT-PCR)

Total RNA was extracted from GC tissues and cells using TRIzol reagent (Waltham, MA, USA) following the manufacturer’s instructions. mRNA levels were assessed by Hifair^®^ III One-Step RT-qPCR SYBR Green Kit (YEASEN, Shanghai, China). All results were normalized to GAPDH, and the relative expression of mRNA was quantified by the 2^–∆∆*Ct*^ method. The required primers are provided in Supplementary Table S2.

### 4.4. Plasmid Construction and Cell Transfection

Human METTL3 and DEK full-length cDNAs were amplified from human cDNA libraries and cloned into the pLVX-EF1α-IRES-Puro vector (Addgene, New York, NY, USA). The sh-RNA targeting METTL3 and DEK were designed and synthesized by Wuhan Qingke Biotechnology (TSINGKE, Beijing, China). MGC80-3 cells or AGS cells were infected with LV-shMETTL3, LV-shDEK, LV-OE-METTL3, LV-OE-DEK and LV-NC, respectively. MGC80-3 cells or AGS cells were induced with 1 μg/mL of puromycin to achieve the stable knockout or overexpression of METTL3 and DEK. The required primers are provided in Supplementary Table S3, and the sh-RNA sequences are provided in Supplementary Table S4.

### 4.5. Western Blot

Total protein was extracted from cells or tissues by lysing cells or tissues in RIPA lysis buffer (Meilunbio, Dalian, China) supplemented with protease and phosphatase inhibitors (Meilunbio, Dalian, China). The protein was quantified by the BCA protein quantification kit. Equal amounts of protein were electrophoresed on 10% or 12.5% SDS-polyacrylamide gels and transferred to polyvinylidene fluoride (PVDF) membranes (Millipore, MA, USA). The PVDF membrane was then incubated in 5% nonfat milk for 1 h at room temperature, then incubated with the specific primary antibody overnight at 4 °C, and the next day, the PVDF membrane was incubated with a horseradish peroxidase-conjugated secondary antibody (Beyotime, Shanghai, China) incubation. The protein bands were observed with the ECL kit. Quantification was conducted using Image J. The primary antibodies used included: anti-METTL3 (CST, 86132, 1:1000, Boston, MA, USA), anti-DEK (CST, 29812, 1:1000, Boston, MA, USA).

### 4.6. Cell Counting Kit-8 (CCK-8) Assay

The proliferation ability of GC cells was evaluated by the CCK-8 method (Beyotime, Shanghai, China). Briefly, 1 × 10^4^ cell suspensions per well were seeded in a 96-well cell culture plate, and the total medium per well was 200 μL. In total, 20 μL of CCK-8 reagent per well was added at 0, 24, 48 and 72 h after transfection, respectively, and the absorbance was measured at 450 nm after incubation at 37 °C for 3 h.

### 4.7. Wound-Healing Assay

GC cells were seeded in 6-well plates, and when the cell confluence reached 90%, a sterile 200 μL pipette tip was used to create wounds in GC cells. Scratch wounds were imaged under an inverted microscope at time points of 0 and 24 h. The average distance between the two edges of the cell-free zone was used to determine wound healing or cell migration rate.

### 4.8. Colony Formation

Cells were seeded into 6-well cell culture plates with 300 cells per well and cultured in a 37 °C incubator for two weeks. Cells were fixed with tissue fixative for 30 min and then stained with 0.1% crystal violet. The colonies of cells formed were counted, and the experiment was repeated in triplicate.

### 4.9. Transwell Assay

For the transwell assay, a chamber with a pore size of 8 μm was used (Corning, 3422, Corning, NY, USA), 5 × 10^5^ cells were added to the upper chamber, resuspended in serum-free medium, and a medium containing 15% fetal bovine serum was added to the lower chamber (the upper chamber side of the polycarbonate membrane was not cover with Matrigel). After 24 h of incubation, the remaining cells in the upper chamber were completely removed, fixed in tissue fixative for 30 min, then stained with 0.1% crystal violet for 20 min, and finally, the cells invading the lower chamber were counted in five fields of view under an inverted microscope.

### 4.10. m6A RNA Dot Blot Assay

RNA was isolated from cells by TRIzol Reagent (CWBIO, CW0580, Beijing, China). RNA was denatured at 65 °C for 5 min and diluted into 40 ng/µL, 20 ng/µL and 10 ng/µL with nuclease-free water. A total of 10 µL of denatured RNA was transferred to nitrocellulose membrane (Biosharp, BS-NC-22, Hefei, China) and crosslinked by UV, and m6A antibody (CST, 56593S, Boston, MA, USA) was incubated. Approximately 0.2% methylene blue (Meilunbio, MB5094, Dalian, China) staining was used as a control.

### 4.11. RNA Immunoprecipitation Assay (RIP)

RIP experiments were performed according to the operating instructions of the RNA Immunoprecipitation Kit (Geneseed, P0102, Guangzhou, China). Briefly, cells were harvested and lysed on ice with lysate, then the sample was incubated with antibody METTL3 (CST, 86132S, Boston, MA, USA) or IgG (ABclonal, AC005, Wuhan, China) (as a control), RNA was extracted after antigen capture and the RNA was analyzed for the expression level of the target gene by qPCR after reverse transcription.

### 4.12. Methylated RNA Immunoprecipitation Assay (MeRIP)

For the MeRIP assay, the Methylated RNA Immunoprecipitation (MeRIP) Kit (BersinBio, Guangzhou, Bes 5203, China) was used. Briefly, the RNA to be analyzed was broken into about 300 bp fragments, and 50 μL was separated as input. The remaining samples were divided into two groups and incubated with m6A and IgG antibodies, respectively (IgG as the control). The enriched RNA was extracted after immunoprecipitation, and qPCR was used for subsequent experimental analysis after RNA reverse transcription.

### 4.13. Dual-Luciferase Reporter Assay

The dual-luciferase reporter gene assay system (Promega, Madison, WI, USA) used the manufacturer’s instructions. The full-length DEK sequence was cloned into a PmirGLO dual-luciferase expression vector (Promega, Madison, WI, USA) containing Renilla luciferase (R-luc) and firefly luciferase (F-luc) to construct a wild-type DEK reporter gene plasmid. To construct DEK mutant reporter plasmids, adenosine groups within the m6A consensus site were replaced with cytosines. WT-DEK, Mut-DEK or negative control plasmids were co-transfected in MGC80-3 and AGS cells, which stably knocked down METTL3. In addition, this study used the Dual-Luciferase report Assay System (Meilunbio, MA0520-1, Dalian, China) to evaluate the activities of F-luc and R-luc 48 h after transfection. Luciferase activity status was normalized using R-luc activity.

### 4.14. BALB/c Nude Mice Animal Models

Lung metastasis model: 1 × 10^5^ luciferase-labeled cells were injected into female BALB/c nude mice (4–5 weeks) via tail vein, and the photon flux in the whole body of the mice was measured using IVIS Lumina Series III (Caliper Life Sciences, Boston, MA, USA). After 28 days, each mouse underwent BLI analysis to monitor lung metastases. All animal experiments were approved by the Laboratory Animal Ethics Committee of Wuhan University of Science and Technology.

### 4.15. IHC Analysis

For immunohistochemistry: sections were deparaffinized and rehydrated; antigens were retrieved with sodium citrate antigen retrieval buffer (pH 6.0), and then endogenous peroxidase was inactivated with 3% hydrogen peroxide for 20 min at room temperature. After washing, nonspecific antigen binding was blocked with 10% normal goat serum for 30 min at 37 °C, incubated with primary antibody overnight at 4 °C and then incubated with secondary antibody for 45 min at room temperature. Chromogenic detection was performed using the 3,3-diaminobenzidine (DAB) chromogenic kit (Servicebio, Wuhan, China). Nuclei were counterstained with hematoxylin. The immunohistochemical results were evaluated as follows: staining intensity was divided into three grades (0 = no staining, 1 = weak, 2 = moderate, 3 = strong), and the percentage of positive areas was divided into four grades (0% (0), <10% (1), 10–30% (2), 31–70% (3), 71–100% (4)). The final score for immunohistochemistry was determined by multiplying the intensity score by the percentage score, with a maximum of 12. Proteins were judged for high (≥6) and low expression (<6) according to the final score.

### 4.16. Bioinformatic Analysis

The TCGA database (https://www.cancer.gov/aboutnci/organization/ccg/research/structural-genomics/tcga, accessed on 24 October 2021) was used to analyze the expression level and prognosis of METTL3, and the UALCAN website (http://ualcan.path.uab.edu/cgi-bin/ualcan-res.pl, accessed on 24 October 2021) was used to further analyze the expression level of METTL3 in GC. The GEPIA database (http://gepia.cancer-pku.cn/, accessed on 26 October 2021) and ENCORI website (https://starbase.sysu.edu.cn/, accessed on 26 October 2021) analysis were used to analyze the correlation between METTL3 and DEK expression. In addition, the SRAMP (http://www.cuilab.cn/sramp, accessed on 26 September 2021) and m6A2Target website (http://m6a2target.canceromics.org/#/, accessed on 27 September 2021) predicted m6A modification of DEK.

### 4.17. Statistical Analysis

All statistics were analyzed using GraphPad Prism 7.0 (GraphPad Software, La Jolla, CA, USA). According to the actual situation, the Student’s *t*-test and one-way analysis of variance test were used. Three independent replicates were performed for each experiment. The value of *p* < 0.05 was considered statistically significant.

## 5. Conclusions

Taken together, this study demonstrates that the m6A methyltransferase METTL3 promotes the proliferation and metastasis of GC through the m6A modification of DEK. The discovery of the METTL3-DEK pathway provides a new direction for the treatment of GC. The discovery of the METTL3-DEK pathway also provides a new target for the treatment of GC.

## Data Availability

The datasets used or analyzed during the current study are available from the corresponding author on reasonable request.

## Supplementary Materials

The following supporting information can be downloaded at: https://www.mdpi.com/article/10.3390/ijms23126451/s1.
