# Supplementary material for: The m6A Methyltransferase METTL3-Mediated N6-Methyladenosine Modification of DEK mRNA to Promote Gastric Cancer Cell Growth and Metastasis
Source: Int J Mol Sci. 2022 Jun 9;23(12):6451. doi: 10.3390/ijms23126451 (PMC9223399; doi:10.3390/ijms23126451)
Supplement: Supplementary file 1 [file ijms-23-06451-s001.zip › ijms-1740068-supplementary.pdf]

Table S1. Relationship between METTL3 expression and clinicopathological characteristics in 20 GC tissues.

Clinicopathologic parameters	n	METTL3 expression		*p*
		low	high	
All cases	20	10	10	
Age				0.738
< 60	6	3	3	
⩾60	14	7	7	
Gender				0.921
male	11	6	5	
female	9	4	5	
TNM stage				0.037
I/II	9	7	2	
III/IV	11	3	8	
Distant metastasis				0.028
No	11	7	4	
Yes	9	3	6	
Lymph nodes metastasis				0.019
No	10	7	3	
Yes	10	3	7	

Table S2. Primer information required for qPCR process.

Primer name	Primer sequence (5′–3′)
qPCR-DEK-Forward	AACTGCTTTACAACAGGCCAG
qPCR-DEK-Reverse	ATGGTTTGCCAGAAGGCTTTG
qPCR-METTL3-Forward	TTGTCTCCAACCTTCCGTAGT
qPCR-METTL3-Reverse	CCAGATCAGAGAGGTGGTGTAG
qPCR-Actin-Forward	CATGTACGTTGCTATCCAGGC
qPCR-Actin -Reverse	CTCCTTAATGTCACGCACGAT
DEK-site1-F	ATTGCACAAGGAAAGGGGCAGA
DEK-site1-R	GCCACTGAACTGACCCACATTCT
DEK-site2-F	ACCATTATACCAGCAAAGAGAAT

DEK-site2-R	ATGCAATTTAAAACAGCAAACTC
DEK-site3-4-F	AACAGTTACCCTGCTTTGCCTC
DEK-site3-4-R	GTGCTTGTACTTAATCCCACCCT
DEK-site5-F	AACAGGTATTTTCACAGGTTTGT
DEK-site5-R	ATCGAAGGCCAATTATTAGGTCT

Table S3. Primer information required for qPCR process.

Primer name	Primer sequence (5′–3′)
DEK-F	ATGTCCGCCTCGGCC
DEK-R	TCAAGAAATTAGCTCTTTTACAGTT
METTL3-F	ATGTCGGACACGTGGAGC
METTL3-R	ATTCTTAGGTTTAGAGATGATACCATCTG

Table S4. Base sequence of shRNA.

shRNA name	targeting sequence
shDEK	GGATAGTTCAGATGATGAA
ShMETTL3	GCCAAGGAACAATCCATTGTT
